# Empathic nonverbal behavior increases ratings of both warmth *and* competence in a medical context

**DOI:** 10.1371/journal.pone.0177758

**Published:** 2017-05-15

**Authors:** Gordon T. Kraft-Todd, Diego A. Reinero, John M. Kelley, Andrea S. Heberlein, Lee Baer, Helen Riess

**Affiliations:** 1 Empathy and Relational Science Program, Department of Psychiatry, Massachusetts General Hospital/Harvard Medical School, Boston, Massachusetts, United States of America; 2 Department of Psychology, Yale University, New Haven, Connecticut, United States of America; 3 Department of Psychology, New York University, New York, New York, United States of America; 4 Program in Placebo Studies, Beth Israel Deaconess Medical Center/Harvard Medical School, Boston, Massachusetts, United States of America; 5 Department of Psychology, Endicott College, Beverly, Massachusetts, United States of America; 6 Department of Psychology, Boston College, Chestnut Hill, Massachusetts, United States of America; Massachusetts College of Pharmacy and Health Sciences, UNITED STATES

## Abstract

In medicine, it is critical that clinicians demonstrate both empathy (perceived as warmth) and competence. Perceptions of these qualities are often intuitive and are based on nonverbal behavior. Emphasizing both warmth and competence may prove problematic, however, because there is evidence that they are inversely related in other settings. We hypothesize that perceptions of physician competence will instead be *positively* correlated with perceptions of physician warmth and empathy, potentially due to changing conceptions of the physician’s role. We test this hypothesis in an analog medical context using a large online sample, manipulating physician nonverbal behaviors suggested to communicate empathy (e.g. eye contact) and competence (the physician’s white coat). Participants rated physicians displaying empathic nonverbal behavior as more empathic, warm, *and* more competent than physicians displaying unempathic nonverbal behavior, adjusting for mood. We found no warmth/competence tradeoff and, additionally, no significant effects of the white coat. Further, compared with male participants, female participants perceived physicians displaying unempathic nonverbal behavior as less empathic. Given the significant consequences of clinician empathy, it is important for clinicians to learn how nonverbal behavior contributes to perceptions of warmth, and use it as another tool to improve their patients’ emotional and physical health.

## Introduction

We define empathy as a social-emotional ability having two distinct components: one *affective*: the ability to share the emotions of others, and one *cognitive*: the ability to understand the emotions of others. This definition is supported by evidence that these two components have dissociable neurological substrates [1, 2]. This approach is broad enough to encompass elements of various components of empathy that have been proposed [3, 4]—e.g. sympathy (or shared emotions) [5], perspective taking [6], and accurate interpersonal perception [7, 8]—while distinguishing basic cognitive processes underlying them. It is necessary for an operational definition of empathy to focus on the cognition of the empathic subject (i.e. the person expressing empathy, such as a physician), but because empathy is fundamentally a relational ability, it is also useful to understand the cognition of the empathic object (i.e. the individual whose emotions are being empathized with, such as a patient). There is extensive evidence that empathy (expressed by the subject) is perceived (by the object) as warmth [e.g. 9]. In this paper, we therefore discuss empathy and warmth as two sides of the same coin (i.e. “empathy/warmth”), in that they are functionally linked in the context of social interaction.

Empathy is particularly important in the context of medicine, where evidence suggests it is related to numerous positive outcomes [for a review, see 10] including increased patient satisfaction [11], good patient rapport [12], increased adherence to treatment [13], increased diagnostic accuracy [14], reduced medical errors [15], and positive health outcomes [16–18] (though see [19]; an intervention of care-giver investment does not alter diabetes outcomes). Other explorations of how physicians are perceived have further differentiated perceptions of empathy, distinguishing two dimensions: caring (similar to high empathy) and dominance (similar to low empathy) [20]. Conveying competence is also important in medicine, as the literature on “medical professionalism” demonstrates [21, 22]. There have even been attempts to institutionalize professionalism through, for example, the doctor’s “white coat” [23]. The white coat has traditionally played a significant part in physician identity formation during medical training [24–28] as well as role identity in clinical settings. Empathy (perceived as warmth) and competence are not only important in medicine, but are two frequently-used dimensions of person perception across many contexts [29].

While ratings of empathy are positively correlated with perceptions of warmth both in the lab [e.g. 30] and in clinical contexts [e.g. 31, 32], there is evidence that perceptions of warmth and competence can be inversely related in some contexts [33–37]. Four mechanisms of the warmth/competence tradeoff are particularly relevant here. First, people can exhibit compensatory judgments of warmth and competence [38] particularly when they are under threat [39]. Because high status and wealth are associated with high ratings of competence [40] and physicians are often considered high status, patients who are threatened by an upward social comparison may be biased to perceive their physicians as less warm. Second, though warmth and competence can be positively related in judgments of individuals, they are more likely to be negatively related in judgments of groups [37]. To the extent that a patient sees a clinician as a member of an outgroup—such as the “upper class,” but also in terms of other social group categories, e.g. gender or ethnicity—they may be more likely to exhibit compensatory judgments of warmth and competence. Third, when comparing two others, people see one individual or group as high on one dimension and low on the other, and the inverse for the object of comparison (e.g. among women [34], businesses [41], and immigrant groups [42]). Patients who are comparing their current clinician experience with others in the past therefore may perceive warm clinicians as less competent and vice versa. Finally, when being perceived, people engage in impression management, downplaying one dimension to highlight the other [36]. Clinicians who are trying to convey empathy, therefore, may downplay their competence.

The field of medicine therefore faces a dilemma: if empathy (perceived as warmth) and competence are both important, but are inversely related in patients’ perceptions, which should be emphasized? Indeed, some have argued that an emphasis on empathic clinician behaviors could negatively impact patient perceptions of clinician competence [43] while others argue that the white coat might emphasize professionalism at the cost of humanism [44, 45]. However, “competence” in the medical context embodies both *technical* competence (i.e. skill in medical procedures and biological knowledge) and *interpersonal* competence (i.e. skill in medical social interactions). Given that patients consider interpersonal competence to be crucial in their evaluations of clinicians, one might expect that competence and empathy/warmth would be positively correlated because empathy is increasingly becoming a component of successful medical care or *interpersonal* competence [46]. This distinction and trend in the understanding of medical competence is consistent with the movement of patient-centered care [47] (and more recently, relationship-centered care [48]), which emphasizes patient experience with and understanding of treatment (including emotional and social implications). Patient- or relationship-centered care models have demonstrated both increased efficiency in treatment and better health outcomes [49, 50]. We therefore predicted that empathic nonverbal behavior will increase perceptions of clinician warmth *and* competence.

Empathy is communicated through both verbal and nonverbal behaviors [51], though the power of nonverbal communication of empathy may be underestimated, as nonverbal behavior can communicate emotional states subtly [52] and automatically [53]. Further, the literature on “thin slicing,” demonstrates that we rapidly make judgments of others [54, 55]. In medical education, emphasis has traditionally been placed on training clinicians in *verbal* communication [56], with relatively little attention paid to *nonverbal* communication [57, 58]. The nonverbal behavior literature provides widespread support for the claim that good nonverbal behavior is crucial to patient-centered care in medicine [59, 60], and identifies a number of specific nonverbal behaviors that influence patients’ perceptions of clinicians [58, 61–63]. For example, open body posture (uncrossed arms), eye contact, smiling, and touch express positive affect, involvement, availability, attention, warmth, encouragement, respect, understanding, empathy, and affiliation with the patient [56, 58, 64–68]. Further, nonverbal communication is also related to positive health outcomes, such as increased pain tolerance [69]. In medical practice, humanistic concern for patient well-being—which can be expressed via these nonverbal behaviors—drives the standard of care forward and incentives for quality care have grown as healthcare reimbursement from third-party payers is now often tied to patient satisfaction surveys [64, 70]. There is relatively less research on nonverbal communication of clinician competence in general [71], and perceptions of the white coat in particular [72]. Because there is a growing debate about the effects of the white coat [68, 73–79], we thought manipulating its presence would be a particularly interesting test of the nonverbal communication of competence.

In the present study, we test whether a warmth/competence trade-off will occur in response to clinicians’ nonverbal behavior. We also test for interactions of participant gender with our independent variables as previous findings have indicated gender differences in judgments of warmth and competence [80–83]. Specifically, while both men and women judge traits related to warmth to be more important than traits related to competence in their formation of impressions of others, women judge the relative importance of traits related to warmth to be significantly more important [84]. There is a gender-role stereotype that women are warmer than men, and these findings might be partially explained by women internalizing this stereotype which transfers to their perception of others [81]. Finally, we wanted to ensure that any effect we found was not driven by mood, such that our manipulations put participants in a positive or negative mood, which then influenced their perceptions of clinicians [85]. Therefore, we included participant mood as a covariate in our analyses to ensure that participants’ ratings of clinicians are attributable to our manipulations. We provide preliminary evidence that nonverbal empathic behaviors increase patient perceptions of clinician empathy, warmth, *and* competence. We find that this effect may be stronger for women, cannot be attributed to mood, and that there is no effect of the white coat.

## Methods

### Participant characteristics

We used the crowdsourcing tool Amazon Mechanical Turk to recruit 1,377 U.S. participants (60% female, 80% white, mean age 36yrs., range 18–80) as analog patients [86] in an online study requiring 7 minutes of participants’ time and paying them $0.30 (commensurate with pay for similar tasks on this platform; see [87–92]). Participants were randomly assigned to 1 of 4 conditions in a 2 (empathic v. unempathic nonverbal behavior) x 2 (white coat v. no white coat) factorial design. This study was run in 2 runs; in the first run (*N* = 194), we did not manipulate the white coat, so subjects were randomly assigned to one of 2 conditions: empathic v. unempathic nonverbal behavior (physicians were wearing the white coat in both conditions). In the second run (*N* = 1,177), we did manipulate the white coat, so subjects were randomly assigned to 1 of 4 conditions in the full 2 (empathic v. unempathic nonverbal behavior) x 2 (white coat v. no white coat) factorial design. The result is that the *white coat* conditions had average *n* = 392 while the *no white coat* conditions had average *n* = 297. We found no significant main effects of run on any of our dependent variables, nor did we find any significant interactions of run with any independent variable, so we collapsed across run for our analyses. The study was IRB-approved by the Partners Human Research Committee and all participants provided online informed consent.

### Study design and protocol

**Fig 1:**
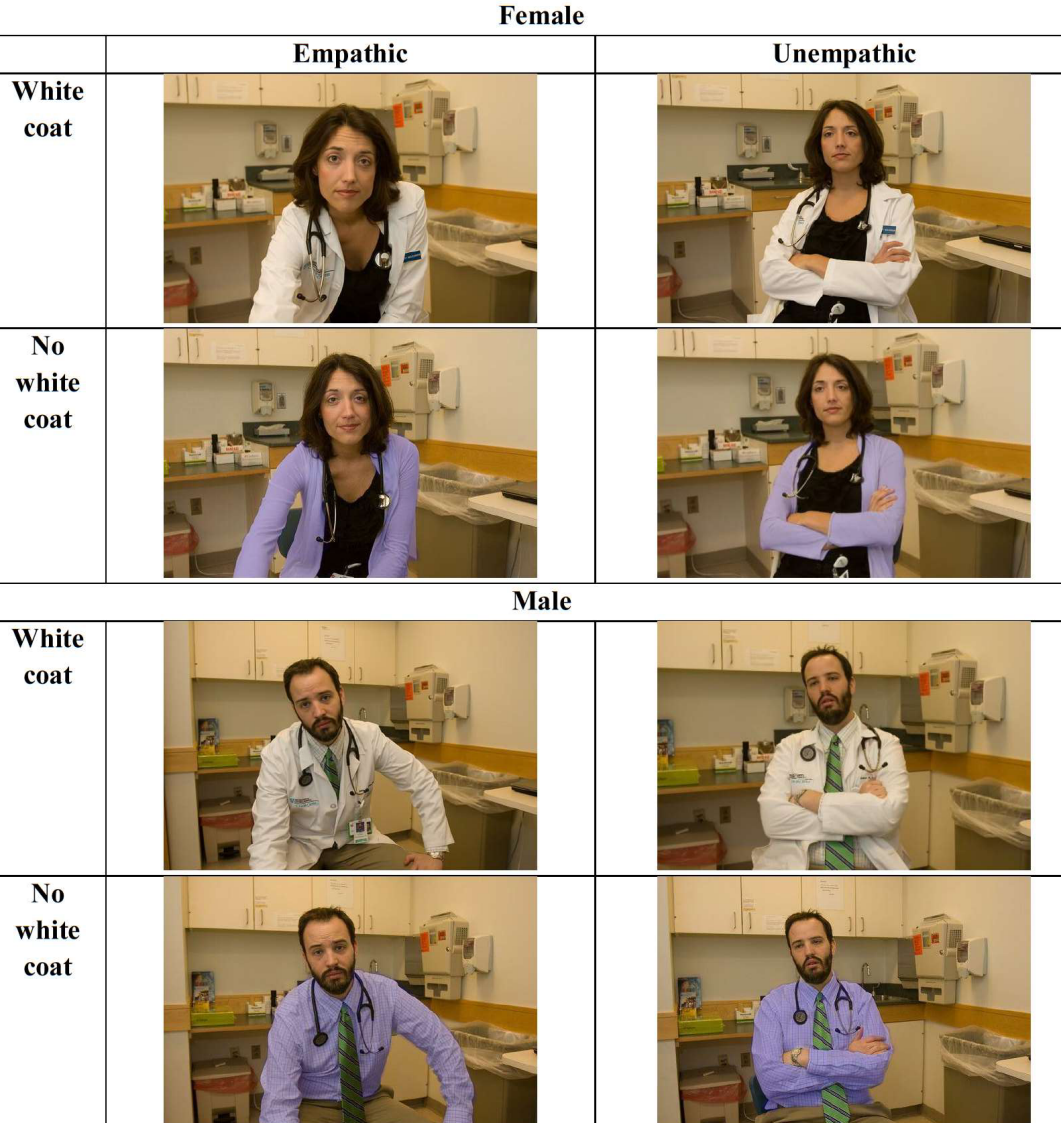
Example photo stimuli (scene 3). Examples of still photograph stimuli used in the experiment depicting a male or female physician displaying empathic or unempathic nonverbal behaviors either wearing or not wearing a white coat.

Participants viewed a series of 6 photographs of either a male or a female (randomly assigned) physician displaying various nonverbal behaviors that were either all *empathic* or *unempathic* (see Fig 1. Photographs were of real physicians—the individuals in these photographs provided written informed consent, as outlined in PLOS consent form, to publish these case details. See S1 File Stimuli for detailed descriptions). Each photograph was paired with 2 lines of a scripted patient-physician conversation (see S1 File Stimuli; all participants saw the same script), and participants were instructed to imagine that they were the patient. In the *empathic* condition, each photograph depicted the same physician displaying various nonverbal behaviors which have been implicated in physician empathy (eye contact, equal patient-physician eye-level, no physical barrier, open posture, touch, and concerned facial expression) [58]. In the *unempathic* condition, the same physician displayed the opposite nonverbal behaviors (no eye contact, unequal eye-level, physical barrier, closed posture, no touch, and unconcerned facial expression). In the *white coat* condition, each photograph depicted the same physician wearing a white coat over their clothes, while in the *no white coat* condition, they simply removed the white coat. All scenes progressed automatically in 3 phases: photograph only (2sec); photograph and script (6sec); photograph only (2sec). This progression allowed participants to focus on the image and nonverbal behaviors displayed by the physician while also incorporating the content of the verbal communication. After viewing all 6 scenes in the interaction, participants completed 3 rating scales (see S2 File Scales) in random order to assess their current mood, perceptions of clinician empathy, and perceptions of clinician warmth and competence. Finally, participants completed several demographic measures, were debriefed, and thanked for their participation.

### Study measures

Mood was measured using the Positive and Negative Affect Scale (PANAS), a 20-item scale with 2 ten-item subscales for positive affect and negative affect (“Indicate to what extent you feel this way right now, at the present moment:” e.g., “Excited”, “Upset”, respectively) rated on a 5-point scale (from “Very slightly or not at all” to “Extremely”) [93]. Both subscales demonstrated high reliability—PANAS positive (α = .90) and PANAS negative (α = .93)—so we created respective composite measures that were the means of the respective items in these subscales. Empathy was measured using the Consultation and Relational Empathy (CARE) measure [66], a 10-item (e.g., “How was the doctor at showing care and compassion (seeming genuinely concerned, connecting with you on a human level; not being indifferent or ‘detached’)?”) 5-point scale (from “poor” to “excellent”). Though there are many measures of empathy in the literature (e.g. [3]), we felt that the widely used, reliable and validated CARE measure was appropriate because it captures the components of our definition of empathy and is tailored to the clinical context. The CARE measure demonstrated high reliability (α = .97) so we created a composite measure that was the mean of the items in this measure. Warmth and competence were measured using a 9-item scale based on Fiske’s stereotype content model [29, 33]; 4 items measured warmth (e.g., “How much did the doctor seem to be caring”) and 5 measured competence (e.g., “How much did the doctor seem to be intelligent”) on a 5-point scale (from 0 to 4). Both measures demonstrated high reliability—warmth (α = .92), competence (α = .71)—so we created respective composite measures that were the means of the respective items in these subscales.

### Data collection and analysis

We used STATA 13.1 to compute all statistics. We found no significant main effects of our white coat manipulation on any of our dependent variables nor did we find any significant interactions of white coat with any independent variable, so we eliminated it from our analyses (and our results are qualitatively the same whether the white coat is included or not). To assess whether physician nonverbal behavior (empathic vs. unempathic) and participant gender affected participants’ ratings of physician empathy, warmth, and competence, we conducted three separate three-way ANCOVAs, with positive and negative mood as covariates.

## Results

### Relation among participant ratings of physician empathy, warmth, and competence

**Fig 2:**
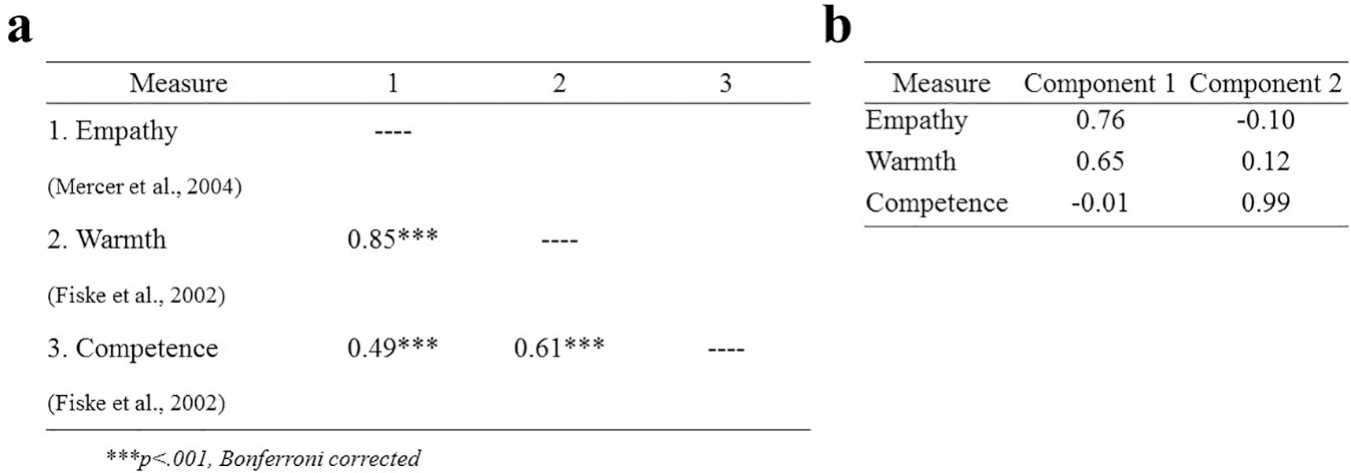
Relation among participant ratings of empathy, warmth, and competence. A) Correlations among the three ratings. B) Principal component weightings of the three ratings on two components using varimax rotation.

We tested for correlations among participant ratings of empathy, warmth and competence (see Fig 2A), and found that each pair was significant (all values shown with Bonferroni corrections) and highly correlated: warmth and empathy (*r*(1375) = .83, *p* < .001), warmth and competence (*r*(1375) = .61, *p* < .001), and competence and empathy (*r*(1375) = .48, *p* < .001). Though we expected a large correlation between empathy and warmth, given the moderate-to-large correlations of competence with empathy and warmth, we performed a principal components analysis with varimax (orthogonal) rotation of these three ratings to determine the extent to which we could interpret competence as a separate rating. We find that two orthogonal factors explain 95% of the variance. We find that while warmth and empathy load strongly on one component, competence loads strongly on the other (Fig 2B).

### Participant ratings of physician empathy

**Fig 3:**
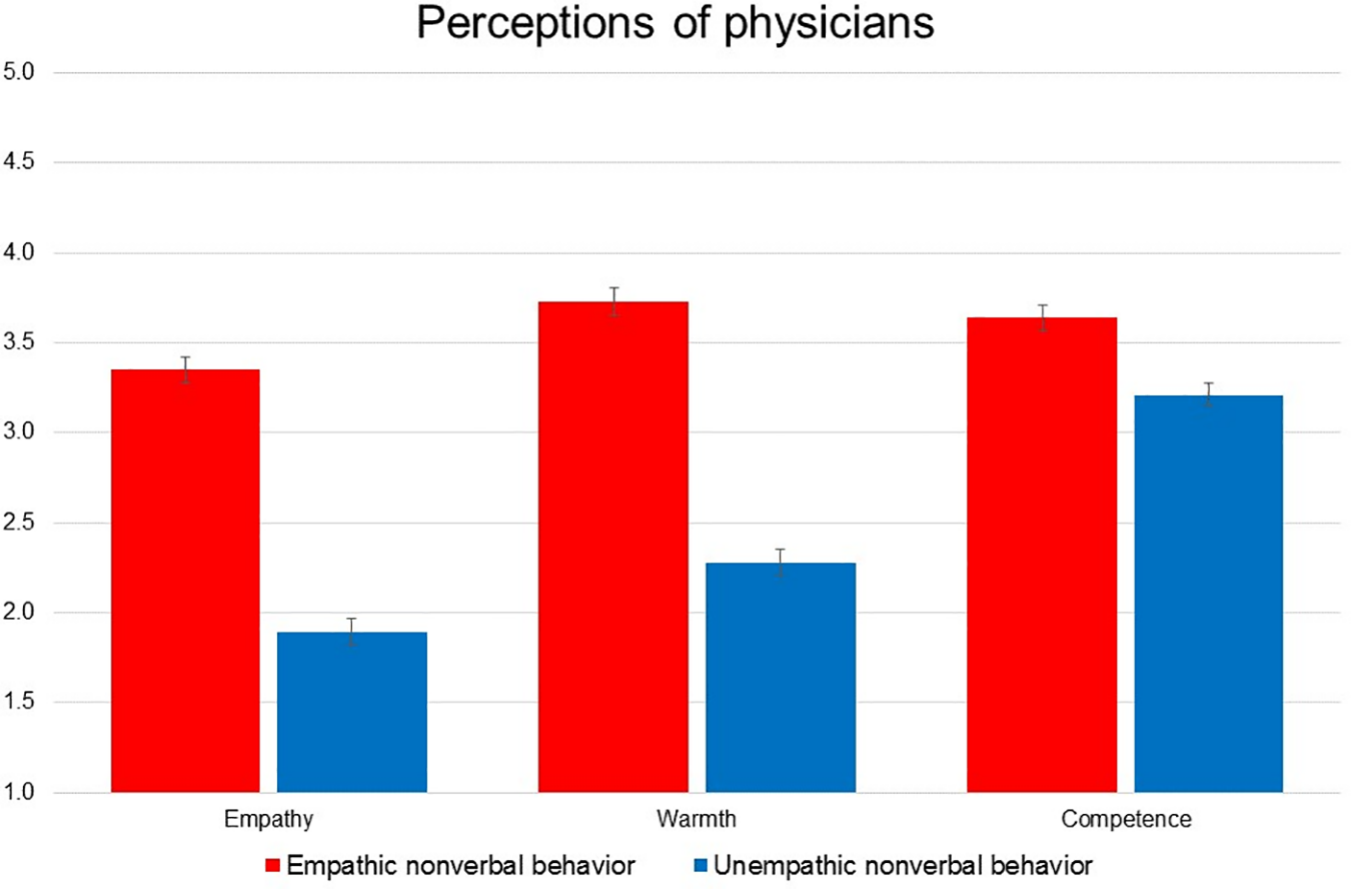
Perceptions of physician nonverbal behavior. Physicians displaying empathic nonverbal behaviors are perceived as more warm, empathic, *and* more competent than physicians displaying unempathic nonverbal behaviors. Error bars represent 95% CI of the mean.

As a manipulation check, we first tested participant ratings of physician empathy. Adjusting for mood, we found a significant main effect of nonverbal behavior on ratings of empathy such that participants rated physicians displaying empathic nonverbal behavior as more empathic (*M* = 3.29, *SD* = 1.15) than physicians displaying unempathic nonverbal behavior (*M* = 1.86, *SD* = .93, *F*(1,1362) = 568.49, *p* < .001, *η*^2^_p_ = .30; see Fig 3). There was also a significant, albeit very small, main effect of subject gender such that male participants rated physicians in both conditions as more empathic (*M* = 2.70, *SD* = 1.21) than the female participants (*M* = 2.49, *SD* = 1.30, *F*(1,1362) = 6.60, *p* = .01, *η*^2^_p_ = .005). There was a marginally significant interaction of nonverbal behavior with participant gender (*F*(1,1362) = 3.00, *p* = .084, *η*^2^_p_ = .002) such that in the unempathic condition, women perceived physicians as less empathic (*M* = 1.73, *SD* = .88) than men (*M* = 2.05, *SD* = .98, *F*(679) = 13.48, *p* < .001, *η*^2^_p_ = .02). Positive mood was a significant covariate of these effects, such that higher positive mood was associated with higher ratings of physician empathy (*F*(1,1362) = 95.98, *p* < .001, *η*^2^_p_ = .07). Negative mood was not a significant covariate of these effects, and there was no association of negative mood with ratings of physician empathy.

### Participant ratings of physician warmth

Adjusting for mood, we found a significant main effect of nonverbal behavior on ratings of warmth such that participants rated physicians displaying empathic nonverbal behavior as more warm (*M* = 3.73, *SD* = .93) than physicians displaying unempathic nonverbal behavior (*M* = 2.28, *SD* = .98, *F*(1,1362) = 674.49, *p* < .001, *η*^2^_p_ = .33). We also found a significant interaction of nonverbal behavior with participant gender (*F*(1,1362) = 4.88, *p* = .027, *η*^2^_p_ = .004) such that in the unempathic condition, women perceived physicians as less warm (*M* = 2.20, *SD* = 0.98) than men (*M* = 2.42, *SD* = .95, *F*(1,679) = 4.46, *p* = .035, *η*^2^_p_ = .007). Positive and negative mood were both significant covariates of these effects, such that higher positive mood was associated with higher ratings of physician warmth (*F*(1,1362) = 64.68, *p* < .001, *η*^2^_p_ = .05) and higher negative mood was associated with lower ratings of physicians warmth (*F*(1,1362) = 12.41, *p* < .001, *η*^2^_p_ = .01).

### Participant ratings of physician competence

Adjusting for mood, there was a significant main effect of nonverbal behavior on ratings of competence, such that participants rated physicians displaying empathic nonverbal behavior as more competent (*M* = 3.64, *SD* = .65) than physicians displaying unempathic nonverbal behavior (*M* = 3.21, *SD* = .81, *F*(1,1362) = 85.11, *p* < .001, *η*^2^_p_ = .06). Positive and negative mood were also significant covariates of these effects, such that higher positive mood was associated with higher ratings of physician competence (*F*(1,1362) = 87.65, *p* < .001, *η*^2^_p_ = .06) and higher negative mood was associated with lower ratings of physician competence (*F*(1,1362) = 14.25, *p* < .001, *η*^2^_p_ = .01).

## Discussion

We provide evidence that nonverbal empathic behaviors increase patient perceptions of clinician empathy, warmth, *and* competence, regardless of whether the clinician is wearing a white coat. Further, this effect cannot be attributed to mood and it may be stronger for women.

In our design, participants were randomly assigned to one of 4 conditions in a 2 (empathic v. unempathic nonverbal behavior) x 2 (white coat or no white coat) factorial design. In the *empathic* nonverbal condition, the physician displayed numerous empathic nonverbal behaviors concurrently, while in the *unempathic* nonverbal condition, the physician displayed numerous unempathic nonverbal behaviors concurrently. In the *white coat* condition, (the same) physicians wore a white coat while in the *no white coat* condition, they did not.

Our manipulation check of whether empathic nonverbal behaviors increased patient perceptions of empathy confirmed our hypothesis, explaining 29% of the variance. Consistent with prior research showing that empathy is perceived as warmth [9], we found empathic nonverbal behaviors increased patient perceptions of warmth, explaining 33% of the variance. Contrary to findings of a warmth/competence tradeoff in other domains [e.g. 34, 36], we found that in the context of patient-clinician interactions, empathic nonverbal behaviors increased patient perceptions of competence, though the effect size was small, explaining 6% of the variance. This might reflect changing expectations about the role of clinicians [64, 67, 94] and the importance of *interpersonal* competence [e.g. 95] in addition to traditional *technical* competence in medical procedures and biological knowledge.

Contrary to prior research [71], we did not find an effect of the clinician white coat on perceptions of empathy, warmth, or competence. Consistent with prior research [96], we found that female subjects were more attuned to signals of empathy than male subjects, as women rated physicians displaying unempathic nonverbal behaviors significantly less empathic and less warm than men, though this effect was quite small, accounting for only 0.3–0.7% of the variance. Finally, mood was a significant covariate of ratings of empathy, warmth, and competence, but it did not explain the effect of our nonverbal behavior manipulation. Positive mood was consistently significantly correlated with our effects, accounting for 5–6% of the variance while negative mood had a smaller effect in the opposite direction, accounting for 1% of the variance.

### Limitations

There are a number of limitations to the current study: 1) null findings from our nonverbal competence manipulation, 2) the online analog design [97], employing still photographs rather than video or live actors, 3) including only one medical context (physicians as the only healthcare provider, both of whom were white, and only one physician of each gender), and 4) clustering empathic nonverbal behaviors without investigating the individual contributions of each. Each of these suggest clear directions for future research.

The null finding of our nonverbal competence manipulation is especially interesting given that the white coat was instituted to convey physician competence [23] and research demonstrating that formal dress increases perception of physician competence [e.g. 98]. One possible explanation for our null result is that it is a “halo effect” [85] of the empathic nonverbal behavior manipulation; i.e., the effect of empathic nonverbal behavior was stronger and participants’ perceptions of empathy influenced their perceptions of competence, overshadowing any effect of the white coat. Other explanations include that the white coat simply does not affect perceptions of physician warmth (and so there might not be as much cause for concern as has been argued [e.g. 75]), or that the analog setting does not evoke ecologically valid reactions to the white coat. Also, it may be that the contrast between competence conditions was not strong enough; in the non-white coat condition, our physicians were wearing semi-formal dress rather than less professional attire, such as jeans and t-shirts (though this would be a manipulation of formal dress rather than the white coat specifically). Further, it should be noted that our design was between-subjects, and so a direct comparison between physicians wearing and not wearing a white coat might make the effect of the white coat more salient, which could be explored in future research using a within-subjects design. Future research might therefore test other manipulations of nonverbal competence behaviors. Finally, though we acknowledge the difference between *interpersonal* and *technical* competence, we did not explore this distinction in the present study. Future research might also disambiguate these components of competence.

This study was conducted online, asking participants to imagine themselves as patients rather than manipulating actual patient-clinician encounters. There is evidence that analog designs can be useful for studies of medical communication [86], though conducting an intervention study in a real medical setting would provide stronger evidence for our conclusions. We did not ask participants whether they thought the physicians pictured were real doctors, which could have affected the extent to which were able to successfully imagine themselves in the scenario. Because manipulations in analog studies could be moderated by the extent of participant engagement, this sort of manipulation check would be important to include in future research. Further, our Amazon Mechanical Turk subject pool is slightly younger, better educated, poorer, and more white than a representative sample of the US population [99] (though more representative than samples drawn from college undergraduates [100]). Finally, by using still photographs, we may be missing subtle cues of nonverbal behavior that are conveyed with more fidelity in video or by using live actors; this is a promising area for future research. While previous research has shown that nonverbal behavior can be explored using photographs [101–104], these more ecologically valid methods would enhance the generalizability of these results.

The medical context we used in this study was intentionally vague; while the script described negative test results and a required surgery (see S1 File Stimuli), much is left unspecified. It is possible that the effect of nonverbal behavior in communicating empathy could be mediated by several situational variables, including medical context (e.g. routine vs. emergency), patient affect (e.g. anxious vs. sad), and the interaction of patient and clinician cultural backgrounds and group identities. Future research could investigate the impact of these situational variables. In particular, there is an important literature using social role theory to explain how perceptions of physician empathy is influenced by physician gender [105–108]. For example, male physicians are evaluated more positively than female physicians when displaying patient-centered behavior, which could be due to the gender-role stereotyped expectation that female physicians will be more patient-centered [109]. In the present study, we did not analyze the effect of physician gender because we were limited to one physician of each gender in our design, and so any physician gender differences might be attributable to the specific physicians we used as models. Future research therefore might use multiple male and female physicians in their stimuli to test for the effect of physician gender on perceptions of empathy/warmth, and competence. In a similar vein, we only investigated the nonverbal behavior of physicians. It is likely that the nonverbal expressions of empathy communicated by other members of a medical team have significant effects on patient outcomes [110], though this question has not received as much research attention.

Finally, we used a “kitchen sink” approach to the nonverbal communication of empathy. In other words, we culled the literature for nonverbal behaviors known to affect perceptions of empathy, and ultimately chose the six that had the most robust evidence base [58]. Our empathic and unempathic conditions varied all six simultaneously, so we were unable to determine the impact of each individual nonverbal behavior on the communication of empathy. Future research could address this question in a deconstruction study that explores patient perception of empathy based on each individual nonverbal behavior, as well as the impact of empathic nonverbal behavior on mood, and the resulting mediating relationship on ratings of empathy/warmth and competence.

### Conclusion

Our findings suggest a clear practical implication: *incorporate empathic nonverbal training into medical education*. A recent meta-analysis shows the empathy trainings can be successful [111], and one in particular emphasizing nonverbal behavior demonstrated a significant increase to patient ratings of clinician empathy [67]. Empathy training is therefore not only possible, but practical, both because third-party payers and the Centers for Medicare and Medicaid Services are increasingly emphasizing patient satisfaction scores for reimbursement and also because the patient-clinician relationship has a positive effect on medical outcomes [16]. Empathy training may even contribute to improved cross-cultural care, as implicit bias against minorities can be communicated nonverbally [112, 113]. Given the significant consequences of clinician empathy, it is important for clinicians to learn how nonverbal behavior contributes to empathic communication, and use it as another tool to improve their patients’ emotional and physical health.

## Supporting information

S1 File Stimuli. Instructions, script, and nonverbal behaviors used as stimuli.
(DOCX)

S2 File Measures. PANAS, CARE, and warmth/competence scales used as dependent measures.
(DOCX)

S3 File Data. Data used for all analyses.
(XLSX)
