# Supplementary material for: Empathic nonverbal behavior increases ratings of both warmth and competence in a medical context
Source: PLoS One. 2017 May 15;12(5):e0177758. doi: 10.1371/journal.pone.0177758 (PMC5432110; doi:10.1371/journal.pone.0177758)
Supplement: S1 File — Instructions, script, and nonverbal behaviors used as stimuli. (DOCX) [file pone.0177758.s001.docx]

**S1 File. Stimuli.** Instructions, script, and nonverbal behaviors used as stimuli.

**Instructions**

You are about to see some pictures of a doctor speaking with a patient.

Imagine that you are the patient, and the doctor is speaking directly to you.

Carefully look at the pictures and read what the doctor and the patient say to one another.

At the end, we will ask you some questions about what you have seen and read.

**Script**

- Scene 1:
  - Doctor: Hello Chris.
  - Patient: Hi.
  - Doctor: So, we’re here to go over your test results together?
  - Patient: I’m really nervous about this.
- Scene 2:
  - Doctor: OK, let’s take a look at the numbers
  - Patient: I really hope everything’s okay.
- Scene 3:
  - Doctor: Unfortunately, these test results show that medical treatment probably won’t work, and I think it would be best if we go through with the surgery.
  - Patient: Oh no, that’s terrible news!  I’ve never had general anesthesia before.
- Scene 4:
  - Doctor: I don’t think there’s anything to worry about; this is a pretty routine procedure.
  - Patient: But aren’t there potential complications?
- Scene 5:
  - Doctor: There is a possibility of complications, but they are relatively rare. I understand your concern, but my team and I have years of experience with this procedure.
  - Patient: So you really think I’ll be okay?
- Scene 6:
  - Doctor: Well, I can’t make any guarantees, but we will take our very best care of you.
  - Patient: OK, I guess we should go ahead.

**Nonverbal Behaviors**

*Note: Empty cells represent behaviors that were not manipulated across condition in the respective scene*

|  | *Scene* | 1 | 2 | 3 | 4 | 5 | 6 |
| --- | --- | --- | --- | --- | --- | --- | --- |
| Eye contact | Empathic | Present | Present | Present | Present | Present | Present |
|  | Unempathic | Absent | Absent | Absent | Absent | Absent | Absent |
| Body posture | Empathic | Open | Open | Open |  |  | Open |
|  | Unempathic | Closed | Closed | Closed |  |  | Closed |
| Body level | Empathic |  |  |  |  | Sitting | Sitting |
|  | Unempathic |  |  |  |  | Standing | Standing |
| Facial expression | Empathic | Concerned |  | Concerned | Concerned |  | Concerned |
|  | Unempathic | Unconcerned |  | Unconcerned | Unconcerned |  | Unconcerned |
| Barrier | Empathic | Absent |  | Absent | Absent | Absent | Absent |
|  | Unempathic | Present |  | Present | Present | Present | Present |
| Touch | Empathic | Extended hand |  | Extended hand |  |  | Extended hand |
|  | Unempathic | None |  | None |  |  | None |
