# Supplementary material for: Empathic nonverbal behavior increases ratings of both warmth and competence in a medical context
Source: PLoS One. 2017 May 15;12(5):e0177758. doi: 10.1371/journal.pone.0177758 (PMC5432110; doi:10.1371/journal.pone.0177758)
Supplement: S2 File — PANAS, CARE, and warmth/competence scales used as dependent measures. (DOCX) [file pone.0177758.s002.docx]

**S2 File. Measures.** PANAS, CARE, and warmth/competence scales used as dependent measures.

*Note: all measures were administered via* Qualtrics *survey software; these are representations of the layout, though the text used was exactly the same*

**PANAS**

*Adapted from Watson et al. 1988*

This scale consists of a number of words that describe different feelings and emotions. Read each item and then mark the appropriate answer in the check boxes next to that word. Indicate to what extent you feel this way right now, at the present moment.

Use the scale below as a reference for all of your answers.

Very slightly or not at all A little Moderately Quite a bit Extremely

Interested O O O O O

Distressed O O O O O

Excited O O O O O

Upset O O O O O

Strong O O O O O

Guilty O O O O O

Scared O O O O O

Hostile O O O O O

Enthusiastic O O O O O

Proud O O O O O

Irritable O O O O O

Alert O O O O O

Ashamed O O O O O

Inspired O O O O O

Nervous O O O O O

Determined O O O O O

Attentive O O O O O

Jittery O O O O O

Active O O O O O

Afraid O O O O O

**CARE**

*Adapted from Mercer et al. 2004*

Poor Fair Good Very good Excellent Does not apply

Making you feel at ease … O O O O O O

(being friendly and warm towards you, treating you with respect, not cold or abrupt)'

Letting you tell your “story” … O O O O O O

(giving you time to fully describe your illness in your own words; not interrupting or diverting you)

Really listening … O O O O O O

(paying close attention to what you were saying; not looking at notes or a computer as you were talking)

Being interested in you as a

whole person … O O O O O O

(asking/knowing relevant details about your life, your situation; not treating you as "just a number")

Fully understanding your

concerns … O O O O O O

(communicating that he/she had accurately understood your concerns; not overlooking or dismissing anything)

Showing care and compassion … O O O O O O

(seeming genuinely concerned, connecting with you on a human level; not being indifferent or “detached”)

Being positive … O O O O O O

(having a positive approach and a positive attitude; being honest but not negative about your problems)

Explaining things clearly … O O O O O O

(fully answering your questions, explaining clearly, giving you adequate information; not being vague)

Helping you to take control … O O O O O O

(exploring with you what you can do to improve your health yourself; encouraging rather than “lecturing” you)

Making a plan of action

with you … O O O O O O

(discussing the options, involving you in decisions as much as you want to be involved; not ignoring your views)

**Warmth/competence**

*Adapted from Fiske et al. 2002*

Please rate each of the following statements about the doctor on the scale from 0 (not at all) to 4 (very much).

How much did the doctor seem to be______________

0 1 2 3 4 Does not apply

Competent O O O O O O

Confident O O O O O O

Independent O O O O O O

Competitive O O O O O O

Intelligent O O O O O O

Tolerant O O O O O O

Warm O O O O O O

Sincere O O O O O O

Good natured O O O O O O
